# Phenolic and Chromatic Properties of Beibinghong Red Ice Wine during and after Vinification

**DOI:** 10.3390/molecules21040431

**Published:** 2016-04-20

**Authors:** Jin-Chen Li, Si-Yu Li, Fei He, Zheng-Yi Yuan, Tao Liu, Malcolm J. Reeves, Chang-Qing Duan

**Affiliations:** 1 Center for Viticulture and Enology, College of Food Science & Nutritional Engineering, China Agricultural University, Beijing 100083, China; jcliwine@hotmail.com (J.-C.L.); 15801203943@163.com (S.-Y.L.); wheyfey@cau.edu.cn (F.H.); 15652347221@163.com (Z.-Y.Y.); mreeves@xtra.co.nz (M.J.R.); 2 Ji’an Municipal Bureau of Agriculture, Ji’an 134200, China; liutao5287@163.com; 3 Institute of Food, Nutrition and Human Health, Massey University, Palmerston North 4442, New Zealand

**Keywords:** Beibinghong red icewine, *V. amurensis* × *V. vinifera* hybrid, phenol, color, vinification

## Abstract

The phenolic and chromatic characteristics of a special red ice wine made from a *Vitis amurensis* × *V. vinifera* hybrid cultivar Beibinghong were studied. Results from two different vintages (2013 and 2014) showed that during vinification, the phenolic acid content increased, while the level of flavonoids (flavonols, flavan-3-ols, and anthocyanins) reduced by a variable extent. The color intensity and red % decreased together with a decrease in anthocyanin content. This was accompanied by an increase in hue as well as yellow %. The final phenolic content was found to be between 119.54 and 180.93 mg/L, with anthocyanins as the predominant phenolic group (92.06%–93.03%), of which 3,5-*O*-diglucosidic anthocyanins made up 53.55%–79.04%. Phenolic acids were the primary non-anthocyanin phenolics at about 6.64%–7.5%. The phenolic contents and color parameters of Beibinghong dry red wine and several *V. vinifera* dry red wines of superior color quality were also used in an attempt to clarify the relationship between phenolics and color in the Beibinghong red ice wine. By using Pearson correlation analysis and principal component analysis (PCA), it was found that 3,5*-O*-diglucosidic anthocyanins and protocatechuic acid were the only characteristic phenolics that differentiated Beibinghong wines from the other selected red wines from more traditional varieties. They were also the main phenolics to be positively correlated with the hue and yellow % of the wine at the early stages leading into maturation. Their presence might, therefore, explain the relatively high hue and yellow % of Beibinghong ice wine.

## 1. Introduction

Phenolic compounds are a group of secondary metabolites in plants which are not only important in protecting plants from diseases and physical wounds, but also in contributing significantly to the sensory and nutritional quality of foods made from fruits and vegetables. The phenolic compounds in red wine contribute greatly to both the organoleptic properties as well as health benefits [1]. Phenolic compounds in wines are divided into two groups according to their chemical structures: flavonoids (including anthocyanins, flavanols, and flavonols) and non-flavonoid phenolics (including hydroxybenzoic acids, hydroxycinnamic acids, and stilbenes) [2]. Thus, different phenolic profiles endow grapes and wines with different qualities [2].

According to their color expression, phenolics in wines can also be classified into two groups by another method: anthocyanins and non-anthocyanin phenolics. Anthocyanins, as a group of important pigments in red grapes and wines, contribute a red to purple color to red wines [3,4]. In young red wines free anthocyanins are the principal source of red color and the relative amounts of the specific anthocyanins determines the exact color of the wines. In red wines made from *V. vinifera* grapes, there are normally 3-*O*-monoglucoside anthocyanins and their acylated derivatives, but in non-*V. vinifera* wines, such as red wines made from *V. amurensis*, *V. davidii*, *V. quinquangularis*, *V. labrusca*, and *V. rotundifolia*, 3,5-*O*-diglucosidic anthocyanins also exist and are usually the predominant anthocyanins [5,6,7,8]. During red wine aging, such free anthocyanins can participate in further reactions to produce other pigments, including pyranoanthocyanins and polymeric anthocyanins, resulting in a gradual color change as they age [9]. Non-anthocyanin phenolics comprised mainly other flavonoids and phenolic acids. Flavan-3-ols, as the main phenolic “skeleton” of the wine, contribute to bitterness and astringency in wines. During aging, polymerization or condensation occurs between flavan-3-ols themselves and other compounds, such as anthocyanins and flavonols, thus altering the wine’s tannin structure and mouthfeel [10]. Flavonols exist in wines mainly in glycosidic forms, the colors of which may range from colorless to yellow. They are responsible for the progressive formation of condensed pigments during the red wine conservation and aging process, which results from the interaction with anthocyanins directly or through acetaldehyde [11]. In addition to contributing astringency and a minor amount of acidity [12], phenolic acids such as hydroxybenzoic acids and hydroxycinnamic acids, can also be involved in oxidizing and browning reactions in wines, and have an impact on the color changes [13,14]. Though such non-anthocyanin phenolics have no red color, they can participate in a complex spontaneous association termed copigmentation with anthocyanins to stabilize and enhance the color expression, essentially in young red wines and, thus, contribute to red wine color, especially in their early maturation.

**Figure 1:**
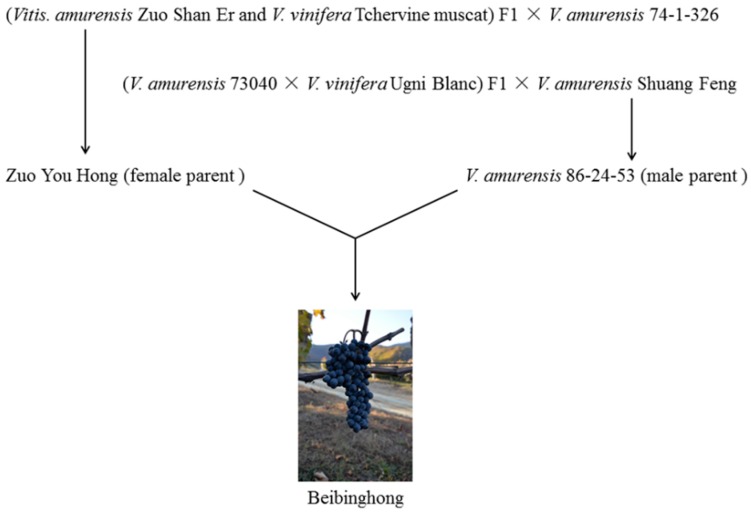
The family tree of hybrid cultivar Beibinghong.

Germany, Austria, and Canada are famous for their prized sweet white ice wines. These are mainly made from some *V. vinifera* cultivars, such as Riesling and Muller Thurgau and, in Canada, Vidal, a French-American hybrid. In recent years great effort has been expended in some other countries in making ice wine and, in particular, developing cultivars suited to ice wine production. In 1995 Beibinghong, a novel hybrid cultivar (*V. amurensis* × *V. vinifera*) was bred in Northeastern China (Figure 1) and after testing was released in 2008. The objective was to achieve both good berry quality and cold tolerance [15]. In Northeastern China, the short frost-free season, extremely low winter temperatures (typically −15 °C) means that the effective accumulated temperature is insufficient for the successful cultivation of *V. vinifera* grapes for ice wine. However, Beibinghong is highly resistant to low temperatures, has medium resistance to some mildew diseases, and yet produces a high yield of good quality grapes. Currently Beibinghong is the world’s first and only cultivar bred for red ice wine. It is widely cultivated in Northeastern China with about 3000 hectares grown mainly for red ice wine. Its products are popular in the Chinese wine market as a local specialty. However, the fact that its color changes to yellow brown relatively quickly (typically within one year), which makes the wine unsuitable for long-term cellaring when compared with *V. vinifera* dry red wines, which are now widely consumed in China. Understandably limited international scientific research has been made into the properties of Beibinghong [15,16]. Current Chinese studies on Beibinghong have been mainly focused on its botanical characteristics, its viticulture, and fruit preservation [17,18].

In order to have a better phenolic and chromatic understanding of this special red icewine and give advice to winemakers based on the phenolic chemistry of the variety, this present study aimed to determine: (1) the phenolic and chromatic change trends of Beibinghong red icewine during vinification and early maturation; and (2) the phenolic reason for the instability of Beibinghong red ice wine’s color.

## 2. Results and Discussion

### 2.1. Sample Preparation and Classification on the Basis of Their Color Quality

Local regional winemaking methods were strictly followed for the vinification of the trial Beibinghong red ice wines of 2013 and 2014. Alcoholic fermentation took 14 days, during which time samples were collected on days 0 (Sampling Point 1), 7 (Sampling Point 2), and 14 (Sampling Point 3). After alcoholic fermentation, the red ice wines were settled in stainless tanks for one month, at which time another sample was taken (Sampling Point 4). More detailed information for the vinification is given in Section 3.1.

Given the well-known instability in color that makes Beibinghong ice wine unsuitable for lengthy maturation, we also chose, for comparison, several dry *V. vinifera* red wines which have good color stability to be included in this study, examining both phenolic compounds and color parameters, the color parameters of different wines will further support our classification for these wines according to their color-stabilities. Additionally, a Beibinghong dry red wine vinified in 2014 was also selected so that, by observing its phenolic and chromatic properties, we might gain a better understanding of the color differences caused by different winemaking techniques. Although these other red wines were to be compared with Beibinghong red ice wine, they were vinified by more normal red wine making methods to produce typical dry red wines. All wine samples were numbered for further study, their information were listed in Table 1. It is pertinent at this point to note that the Beibinghong ice wine is not fermented on skins, unlike the other wines.

In order to confirm the wine quality we referred to as color-unstable and color-stable in Table 1, color parameters of each final wine were analyzed and are listed in Table 2. As shown in Table 2, Beibinghong red ice wine (BH-IW13 and BH-IW14) had the lowest color intensity and red % values, and also the highest hue and yellow % values among all wine samples. Beibinghong dry red wine (BH-DW14) had the highest color intensity, but its red %, hue and yellow % values were almost of the same magnitude as that of the Beibinghong ice wine (BH-IW13 and BH-IW14). It is well known that when a red wine ages it changes from a purple red when young to a brick red when mature. Chromatically the red % reduces and the yellow % increases. Of the seven samples tested, the Beibinghong wines (BH-IW13, BH-IW14 and BH-DW14) had the lowest red % and highest yellow % values and also the highest hue which corresponds to the highest yellow brown color, which meant that their color were easier to degrade than the wine color of other *V. vinifera* dry red wines we chose.

### 2.2. Identification of Phenolics and the Overall Quantitative Analysis

Considering the importance of phenolics, especially anthocyanins, to wine color, the composition and concentration of the different phenolics in red wine is likely to be the main cause of color differences. By examining the phenolic composition of the seven wines it should be possible to determine a possible reason for the rapid color development in the Beibinghong wine. The phenolic compounds in the red ice wines and *V. vinifera* dry red wines were mainly identified by their MS/MS information (Table 3) and UV absorption (anthocyanins: 525 nm; non-anthocyanin phenolics: 280 nm), and their quantifications were done only by their UV absorption, which were all according to our previously published studies [19].

In Table 3, a total of 54 phenolic compounds were detected and identified. Of these, 37 phenolics were detected in the ice wine and 25 were quantified. The Beibinghong wine made in 2013 (BH-IW13) had a higher phenolic content than the Beibinghong ice wine made in 2014 (BH-IW14). However, the differences in proportions of each phenolic class were quite small. At 93.03% in the 2013 and 92.06% in the 2014 wine, anthocyanins are the dominant class of phenolics in both ice wines. The remainder is principally the phenolic acids, hydroxybenzoic and hydroxycinnamic, comprising 6.64% and 7.5% for the 2013 and 2014 vintage wines, respectively. Of the minority groups of polyphenols, flavonols comprised just 0.33% in the 2013 and 0.44% in the 2014 wines while flavan-3-ols were either in trace amounts or not detected. The total non-anthocyanin contents were just 12.61 mg/L and 9.49 mg/L for the 2013 and 2014 wines, respectively, of which phenolic acids accounted for a very high proportion at 12.01 mg/L and 8.97 mg/L.

#### 2.2.1. Anthocyanins

As shown in Table 3 a total of 20 anthocyanins were identified in the Beibinghong icewine, including 5 3,5-*O*-diglucosidic anthocyanins and 15 3-*O*-monoglucosidic anthocyanins. Of these the 3,5-*O*-diglucosidic anthocyanins were the predominant anthocyanins, being 53.55% (BH-IW13) and 79.04% (BH-IW14) of the total anthocyanins. This was in accord with the experience that 3,5-*O*-diglucosidic anthocyanins usually dominate the anthocyanins profile of *V. amurensis* grapes and their hybrids, as well as their wines [7]. Though pelargonidin-based anthocyanins were reported in some *V. amurensis* grapes and wines previously [7], and in some Concord (*V. labrusca*), Rubired (*V. vinifera* × *V. rupestris*), and Salvador (*V. vinifera* × *V. rupestris*) grape juice [20], no quantitative information for pelargonidin-based anthocyanins has been found in Beibinghong red ice wines, which might in part be due to their limited presence and maceration time since, in Beibinghong dry red wine (BH-DW14), a trace amount of pelargonidin-3,5-*O*-diglucoside was found.

#### 2.2.2. Phenolic Acids

In the Beibinghong ice wine we detected five hydroxybenzoic acids namely, 2-hydroxybenzoic acid, gallic acid, protocatechuic acid, vanillic acid, and syringic acid. However, in some *V. amurensis* dry red wines, 2-hydroxybenzoic acids and vanillic acid were not detected, while ethyl gallate and ellagic acid has been found in *V. amurensis* dry red wines [21] but they were absent in the ice wines, possibly due to the limited contribution of skins and seeds to the phenolic composition in ice wines. The proportion of hydroxybenzoic acids of ice wines BH-IW13 and BH-IW14 in non-anthocyanin phenolics were 67.01% and 94.52%, respectively. In Beibinghong dry red wine (BH-DW14), the hydroxybenzoic acids proportion of the non-anthocyanin phenolics was 50.57%. In the color-stable red wine samples (ME-DW13, CG-DW13, MA-DW13, CS-DW13), the hydroxybenzoic acids ratio in non-anthocyanin phenolics ranged from 12.46%–16.23%.

As involved in wine co-pigmentation, hydroxycinnamic acids usually play vital roles in the oxidative discoloration of wine, as well as in the non-enzymatic process of hydroxyphenyl-pyranoanthocyanin formation [22]. In research into ice wine, 3 hydroxycinnamic acids were detected in the final product, being 4-hydroxycinnamic acid, caffeic acid and ferulic acid. In comparison *V. amurensis* dry red wines had four more hydroxycinnamic acids, *trans*-caftaric acid, *trans*-coutaric acid, *trans*-fertaric acid, and ethyl caffeic acid [21]. The proportion of hydroxycinnamic acids in the non-anthocyanin phenolics of the icewine was 28.23% in BH-IW13, but only a trace in BH-IW14. In Beibinghong dry red wine (BH-DW14), the hydroxycinnamic acids proportion of the non-anthocyanin phenolics was 10.52%. Regardless of proportion difference, the content difference between Beibinghong red ice wine and dry red wine could well be the result of the pressing of frozen grapes for ice wine *vs.* on-skins fermentation for normally dry red wines. In color-stable red wines (ME-DW13, CG-DW13, MA-DW13, CS-DW13), the hydroxycinnamic acid proportion of the non-anthocyanin phenolics ranged from 0.36%–3.29%.

#### 2.2.3. Flavan-3-ols

Only a trace amount of procyanidins B1 and B2 were detected in the ice wines. In other *V. amurensis* dry red wines, the content of flavan-3-ols ranged from 2.38 to 46.74 mg/L, with (+)-catechin, (−)-epicatechin, procyanidin B1, procyanidins B2 and C1 being reported [21]. In the Beibinghong dry red wine (BH-DW14), we found a flavan-3-ol content of 17.62 mg/L, and the flavan-3-ols made up 16.04% of the non-anthocyanin phenolics. In the color-stable red wines (ME-DW13, CG-DW13, MA-DW13, CS-DW13), more flavan-3-ols were detected, which were (+)-catechin, (−)-epicatechin, gallocatechin, epicatechin gallate, procyanidin B1, B2, and C1, resulting in the flavan-3-ols percentage in non-anthocyanin phenolics ranging from 53.82%–64.88%.It is well known that the different forms and amounts of flavan-3-ols present in grape skins and seeds impacts on the bitter and astringency mouth feel of red wines. The extremely low levels of flavan-3-ols in the Beibinghong ice wine means that these will make only a limited contribution to these taste parameters, making it different from the regular dry red wines made from *V. vinifera* and *V. amurensis* grapes.

#### 2.2.4. Flavonols

The presence of flavonols in the ice wine also showed a great disparity with those of *V. vinifera* and *V. amurensis* dry red wines. The percentage of flavonols in non-anthocyanin phenolics of the icewine was 4.76% (BH-IW13) and 5.48% (BH-IW14) and seven flavonols (dihydrokeampferol, dihydroquercetin, quercetin-3-*O*-glucoside, quercetin-3-*O*-galactoside, quercetin-3-*O*-glucuronide, myricetin-3-*O*-glucoside, and syringetin-3-*O*-glucoside) were found at low levels. It has been reported that naringenin, luteolin, quercetin, and myricetin are present in *V. amurensis* dry red wines as well as the seven flavonols that we found in the ice wine, giving total levels ranging from 4.75 mg/L to 23.93 mg/L [21]. In Beibinghong dry red wine (BH-DW14), the flavonols proportion of non-anthocyanin phenolics was 22.87%. As for the color-stable red wines we chose (ME-DW13, CG-DW13, MA-DW13, and CS-DW13), their flavonol percentages of non-anthocyanin phenolics ranged from 19.37%–28.31%.

Compared with dry red wines from Beibinghong and *V. vinifera*, the relatively low phenolic level in Beibinghong ice wine might be due to the special enological characteristics, principally the pressing of frozen grapes. Without the maceration process, phenolic compounds would not be extracted from the berry skins and seeds into the grape juice easily and abundantly, and only limited phenolics would transferred during pressing of the frozen berries [23,24].

### 2.3. Evolution of Phenolic and Chromatic Characteristics of Beibinghong Red Ice Wine during Vinification

**Figure 2:**
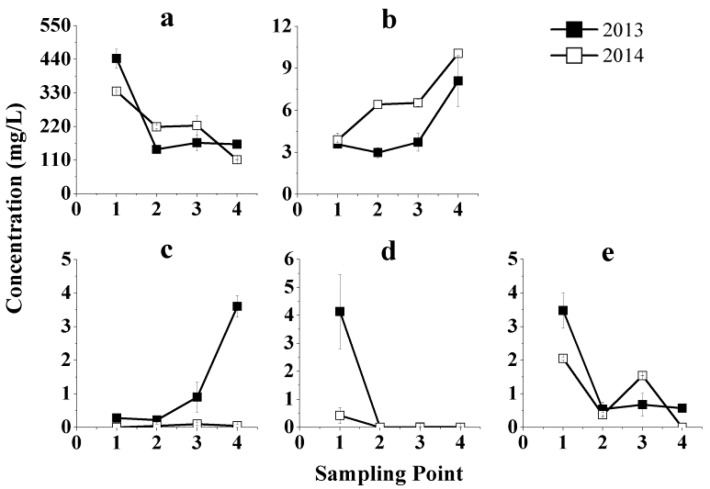
Phenolic evolution during vinification of Beibinghong red ice wine from two vintages, 2013 and 2014. (**a**) Evolution of anthocyanins during vinification; (**b**) Evolution of hydroxybenzoic acids during vinification; (**c**) Evolution of hydroxycinnamic acids during vinification; (**d**) Evolution of flavan-3-ols during vinification; (**e**) Evolution of flavonols during vinification.

The evolution of the five groups of phenolic compounds in Beibinghong ice wine are shown in Figure 2. It is clear that only the phenolic acids, hydroxybenzoic acids (Figure 2b) and hydroxycinnamic acids (Figure 2c) increased during vinification. The other three groups of phenolic compounds, namely, anthocyanins, flavan-3-ols, and flavonols all fell to varying degrees. The concentration of flavan-3-ols fell to non-detectable concentration levels during vinification (Figure 2d), and anthocyanins showed the biggest drop quantitatively. Naturally, this would have a serious impact on the wine color intensity and hue. Since no maceration was done during vinification, the total phenolic content of the juice was already at a maximum level after pressing as the grape pomace was separated from the juice after pressing and phenolic compounds in skins or seeds could not be further extracted. Some individual phenolics would be lost by degradation, clarification and absorption by yeast, such as the anthocyanins and other flavanoids, but others, such as the phenolic acids, might increase due to ester hydrolysis.

**Figure 3:**
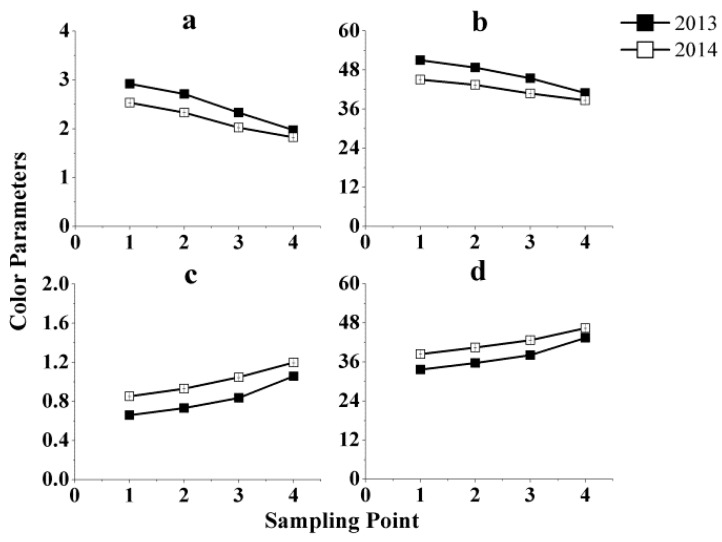
Chromatic evolution during vinification of Beibinghong red icewine from two vintages, 2013 and 2014. (**a**) Evolution of color intensity during vinification; (**b**) Evolution of red % during vinification; (**c**) Evolution of hue during vinification; (**d**) Evolution of yellow % during vinification.

Figure 3 shows the evolution of the four major color parameters of the two red ice wines, 2013 and 2014, during vinification. The trends for both years for color intensity and red % were similar, while the same is true for the hue and yellow %. The values of color intensity and red % fell as a whole, and the extent varied during different stages of vinification. The drop in color intensity followed a similar trend in phenolics as shown in Figure 2, especially with the changes in anthocyanins, which is to be expected given that the anthocyanins made up the largest proportion of phenols. The decrease of all the phenolic compounds related to the color of the wine is in line with increase to the values of hue and yellow %. It is normal for the hue of wine to increase and a shift in hue from bright red to red-brown hue after the level of anthocyanin compounds had peaked and this occurs especially when there is no maceration. This can be explained by the formation of pigmented polymers and also the formation of brown pigments resulting from phenolic oxidation [25,26]. Moreover, on the basis of the fact that the content of flavonols and flavan-3-ols were decreasing as well, it may be deduced that the increase in hue and yellow % values could be the combined result of decreases in anthocyanins, flavonols, and flavan-3-ols, and increase in phenolic acids. Judging from Figure 3, we expect that the color development of the red ice wine will become increasingly unacceptable to consumers.

### 2.4. Statistical Analysis for Key Phenolic Compounds that Affect Color Quality of Beibinghong Red Ice Wine

**Figure 4:**
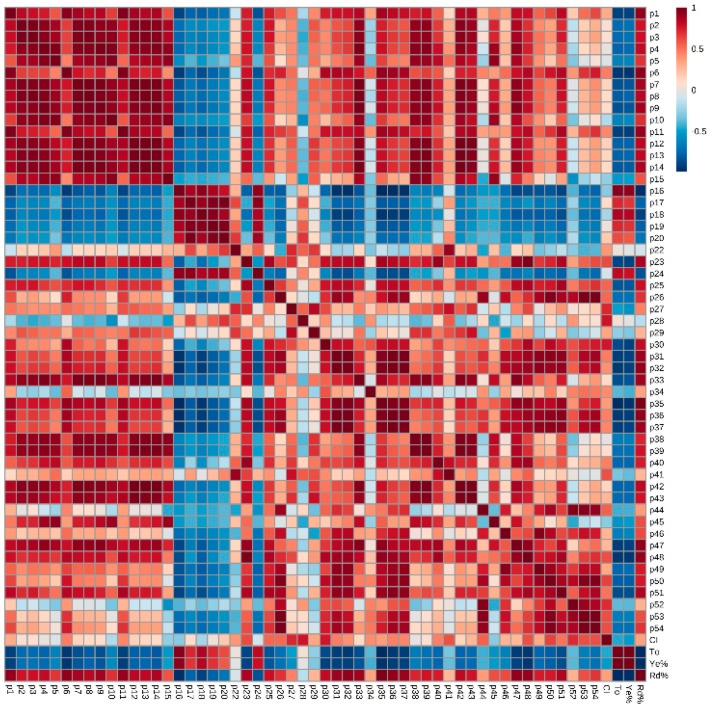
Pearson correlation analysis between phenolic compounds and color parameters.

**Figure 5:**
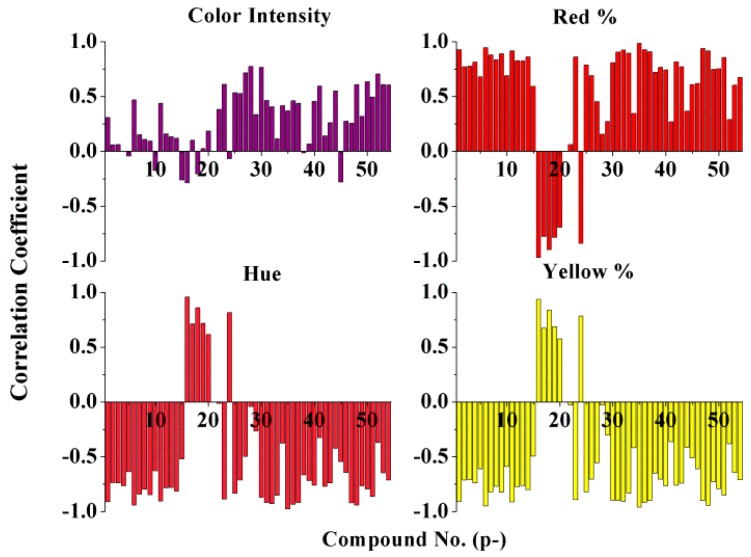
Correlation coefficients of color parameters with phenolic compounds.

In order to find out the phenolic compounds that played a determinant role in the final ice wine’s color quality, Pearson correlation analysis (Figure 4) was used to establish the relationship between phenolic compounds and color parameters on the basis of phenolic and chromatic data of wine samples listed in Table 1. The correlation values for the phenolic compounds with the color parameters (Figure 5) show that color intensity is positively related to all phenolics, except cyanidin-3-*O*-glucoside (p5), cyanidin-3-*O*-acetylglucoside (p10), cyanidin-3-*O*-coumaroylglucoside (p15), malvidin-3,5-*O*-diglucoside (p16), delphinidin-3,5-*O*-diglucoside (p18), protocatechuic acid (p24), kaempferol(p38), and kaempferol-3-*O*-galactoside (p45), while red % was positively related to all phenolics except malvidin-3,5-*O*-diglucoside (p16), petunidin-3,5-*O*-diglucoside (p17), delphinidin-3,5-*O*-diglucoside (p18), peonidin-3,5-*O*-diglucoside (p19), cyanidin-3,5-*O*-diglucoside (p20) and protocatechuic acid (p24); hue and yellow % were negatively related to all phenolics except malvidin-3,5-*O*-diglucoside (p16), petunidin-3,5-*O*-diglucoside (p17), delphinidin-3,5-*O*-diglucoside (p18), peonidin-3,5-*O*-diglucoside (p19), cyanidin-3,5-*O*-diglucoside (p20), and protocatechuic acid (p24).

**Figure 6:**
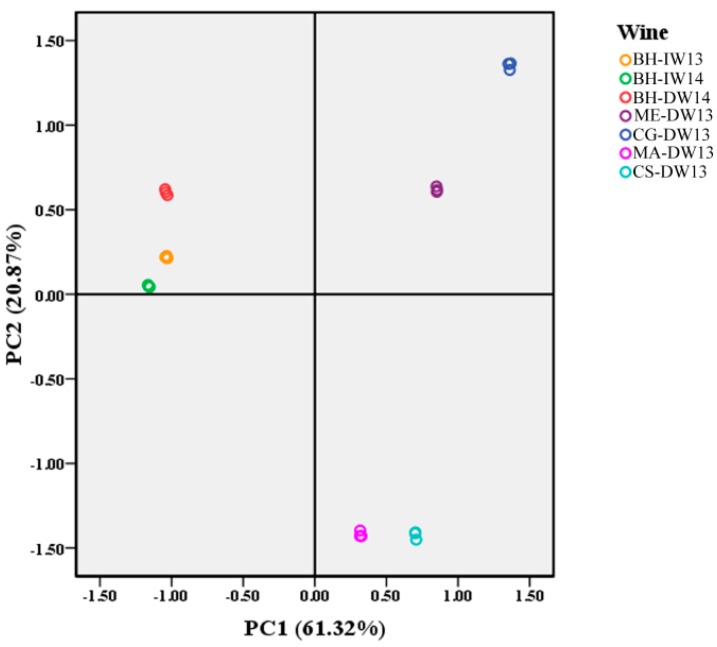
PCA analysis of phenolic compounds in different wine products.

**Figure 7:**
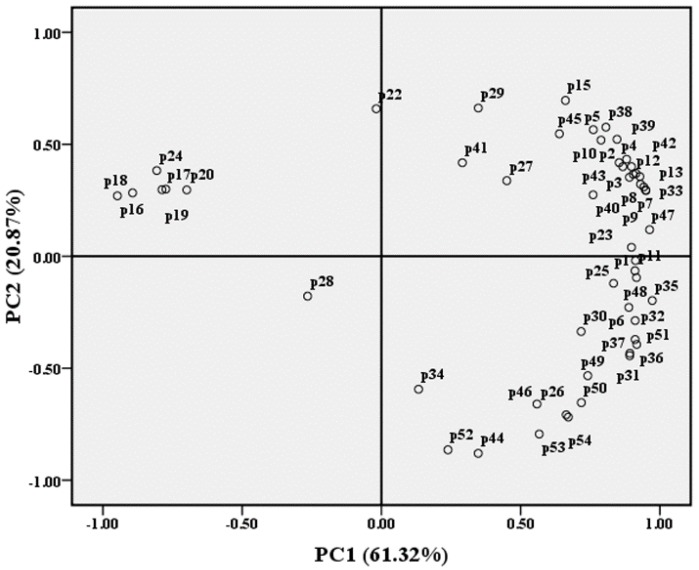
Loading plots of different phenolic compounds in PC1 and PC2.

By using principal component analysis (PCA) to differentiate the wine samples listed in Table 1, we found that the Beibinghong wines (both ice wine and dry red wine) were, to a large extent, separated from the four color-stable *V. vinifera* dry red wines by PC1 (Figure 6). Considering the loading plots of different phenolic compounds in PC1 (Figure 7), it was obvious that malvidin-3,5-*O*-diglucoside (p16), petunidin-3,5-*O*-diglucoside (p17), delphinidin-3,5-*O*-diglucoside (p18), peonidin-3,5-*O*-diglucoside (p19), cyanidin-3,5-*O*-diglucoside (p20), and protocatechuic acid (p24) were the only characteristic phenolics that separated Beibinghong wines from the four color-stable red wines, for the reason that Beibinghong wines located in the top left quadrant of the PCA score plot (Figure 6) showed high positive correlation to these six phenolic compounds located in the top left quadrant in the PCA loadings plot (Figure 7).

Combining the results from Pearson correlation analysis and PCA, we can conclude that the abundance of malvidin-3,5-*O*-diglucoside (p16), petunidin-3,5-*O*-diglucoside (p17), delphinidin-3,5-*O*-diglucoside (p18), peonidin-3,5-*O*-diglucoside (p19), cyanidin-3,5-*O*-diglucoside (p20), and protocatechuic acid (p24) and the relatively low amount of other phenolics in Beibinghong ice wine should, to some extent be related to this special ice wine’s unstable color. This is in contrast to reports that 3,5-*O*-diglucosidic anthocyanins are more stable than their 3-*O*-monoglucosidic counterparts, but are more susceptible to browning and are less colored at wine pH [27,28,29]. This made be due to the fact that some non-anthocyanin phenolics that can help improve color stability, were found in trace or low amount in Beibinghong wines, but were abundant in color stable *V. vinifera* dry red wines. For instance, (+)-catechin can play a significant role in the development and stabilization of color through the acetaldehyde-mediated condensation between (+)-catechin and anthocyanin-3-*O*-glucosides [30]. It is also easily and maybe preferentially oxidized in most red wines thereby protecting other oxidizable compounds, including anthocyanins and their early colored polymers, from oxidation and, thus, helping stability [31]. The flavonol compounds, which were abundant in the color stable wines but which were relatively low in Beibinghong wines, may be involved in copigmentation. Copigmentation can enhance early wine color intensity, hue, and stability [32]. While the special vinification techniques used for Beibinghong red ice wine, in particular the lack of an on-skins fermentation step would lead to a low level or absence of many phenolic compounds in Beibinghong red ice wine, the phenolic contents in Beibinghong dry red wine were still much lower than other color stable *V. vinifera* dry red wines. The low phenolic levels would be determined by the phenolic synthesis genes of this grape variety.

## 3. Materials and Methods

### 3.1. Samples and Vinification

Frozen Beibinghong grapes were harvested in 2013 and 2014 from a vineyard located in Shihu village (126°32′22′′E, 41°22′15′′N), Ji’an City, Jilin Province in Northeast China. The Beibinghong vines were grafted onto “Beta” rootstock and planted in 2010 in a north-south row orientation. All the vines were trained to VSP system, at a 2.5 m × 0.8 m spacing. The vineyard of Ji’an City has a temperate continental monsoon climate, characterized by four seasons, high biologically-effective day temperatures (ranging from 23 April to 23 October, 2013, 27 March to 1 November, 2014), a large temperature difference between daytime and nighttime (there is an average maximum difference of 18.1–19.8 °C in July), and medium annual rainfall (4.97 mm in August and September, 2013, 2.29 mm in August and September, 2014). The annual average sunlight hours during the growing season was 6 h in 2013 and 7 h in 2014, annual average temperature 7.2 °C (2013) and 5.5 °C (2014). All of the meteorological data of 2013 and 2014 were obtained from the local meteorological administration [33].

The Beibinghong grapes were picked at night when the fruits were fully frozen due to the temperature dropping rapidly to about −20 °C. At picking, the pressed juice of the grapes had a total acidity 13.35 g/L (2013) and 14.5 g/L (2014), tartaric acid equivalents, pH 3.19 (2013) and 3.4 (2014), 28.9 Brix (2013), and 37.8 Brix (2014). The frozen berries were pressed in a special vertical press, and then 90 mg/L SO_2_ and 50 mg/L pectinase (Zym Extra, Enartis, Trecate, Italy) were added. The clarified juice was then transported at a low temperature to the winery of the Center for Viticulture and Enology of China Agricultural University in Beijing. Then, another 60 mg/L pectinase (Zym Extra, Enartis) was added to the juice, and clarified with bentonite (500 g/m^3^, Incanto, Enartis). During the process of clarification, the temperature was kept under 5 °C. After a week of clarification, 0.4 g/L activated dry yeast (Red fruit, Enartis) was reactivated and added to the juices to start the alcoholic fermentation at under 13 °C. Three 2000 L fermenters were used. The alcoholic fermentation was terminated after 14 days when the alcohol level reached 10% (*v*/*v*). During alcoholic fermentation, wine samples were collected separately when the specific gravity decreased by 0.02 at each step. This corresponded to collecting samples at day 0 (Sampling Point 1), 7 (Sampling Point 2), and 14 (Sampling Point 3). When the alcoholic fermentation was terminated, the red ice wines were preserved in stainless tanks for one month, and another sample collected was performed at the end of tank preservation (Sampling Point 4). Four samples were collected from each of the three fermentations. The collected samples were centrifuged at 8000 rpm at 4 °C for ten minutes, the supernatants were collected and stored at −20 °C for analysis.

To help establish the phenolics responsible for the color characteristics and the changes during early maturation of the Beibinghong red icewines, four representative *V. vinifera* dry red wines that were known to have good color stability were chosen for comparison. These were Merlot, Cabernet Gernischt, Marselan, and Cabernet Sauvignon, which had all been vinified in CiticGuoan Winery, Xinjiang Province in 2013. Additionally, a Beibinghong dry red wine vinified in Tong Tian Winery, Jilin Province in 2014 was also selected. By observing their phenolic and chromatic properties, we wish to have a better understanding on the color differences caused by different winemaking techniques. The winemaking procedures used strictly followed the local winemaking standards.

### 3.2. Chemicals and Standards

The standard, malvidin-3-*O*-glucoside was purchased from Extrasynthese SA Co. (Lyon, France), while gallic acid, caffeic acid, (+)*-*catechin, and quercetin were all purchased from Sigma Chemical Co. (St. Louis, MO, USA). Methanol, formic acid, acetic acid, and acetonitrile (HPLC grade) were obtained from Fisher Co. (Fairlawn, NJ, USA). Ethyl acetate (analytical grade) was from Beijing Chemical Reagent Plant (Beijing, China).Water was prepared from a Milli-Q system (Molsheim, France).

### 3.3. Determination of Chromatic Characteristics of Wines

A spectrophotometer (Shimadzu, Tokyo, Japan) was used to record the wines’ absorbance spectra (200–780 nm) in a 1 mm path length quartz cuvette. The scanning speed was 2400 nm/min. Using absorbance values at 420, 520, and 620 nm, four colorimetric parameters were calculated as described by Glories [34]. These parameters were color intensity (CI), hue, red % and yellow %, and were calculated according to Equations (1)–(4):

(1) $$\text{CI}={\text{Abs}}_{420}+{\text{Abs}}_{520}+{\text{Abs}}_{620}$$

(2) $$\text{Hue}={\text{Abs}}_{420}/{\text{Abs}}_{520}$$

(3) $$\text{Red }\%=\left(\frac{{\text{Abs}}_{520}}{\text{CI}}\right)\times 100\%$$

(4) $$\text{Yellow }\%=\left(\frac{{\text{Abs}}_{420}}{\text{CI}}\right)\times 100\%$$

Each analysis was performed in triplicate.

### 3.4. Extraction of Phenolic Compounds

The extraction and HPLC-MS/MS analysis methods for the phenolic compounds used were those described by Li *et al*., with some modifications [19]. For anthocyanins, a Beibinghong wine sample of 2 mL was diluted with distilled water by five times to reduce the viscosity and 1 mL diluted sample was filtered using 0.45 μm polyether sulphone membranes, then injected directly into the HPLC-MS/MS. The *V. vinifera* dry red wines were analyzed with the same procedure but without dilution.

For non-anthocyanin phenolics (including hydroxybenzoic acids, hydroxycinnamic acids, and flavan-3-ols and flavonols), a wine sample of 100 mL was diluted with the same volume of distilled water and then extracted three times with ethyl acetate (80 mL). The different ethyl acetate extracts were combined and the pooled extracts were evaporated to dryness by a rotary evaporator at 30 °C; the remainder was re-dissolved in methanol (chromatography grade) up to a final volume of 5 mL. The final sample was filtered by using 0.22 μm organic membranes (nylon) prior to analysis by HPLC-MS/MS.

### 3.5. Quantitative Analyses by HPLC-MS/MS

For anthocyanins, an Agilent 1100 series LC-MSD trap VL equipped with a G1379A degasser, a G1311A pump, a G1313A auto liquid sampler (ALS), a G1316A column compartment, a G1315B diode array detector (DAD) and a Kromasil-C18 column (250 × 4.6 mm, 5 μm), was used. The mobile phase was composed of (A) 6% (*v*/*v*) acetonitrile containing 2% (*v*/*v*) formic acid, and (B) 54% (*v*/*v*) acetonitrile containing 2% (*v*/*v*) formic acid. The gradient elution was as follows: 10% B for 1 min, from10% to 25% B for 17 min, isocratic 25% B for 2 min, from 25% to 40% for 10 min, from 40% to 70% for 5 min, and from 70% to 100% for 5 min, with a flow rate at 1.0 mL/min. The injection volume was 30 μL and the detection wavelength was 525 nm. The column temperature was 50 °C. The MS conditions were as follows: electrospray ionization (ESI), positive ion model; nebulizer, 35 psi; dry gas flow, 10 L/min; dry gas temperature, 325 °C; scan, 100–1000 *m/z*.

For the non-anthocyanin phenolic compounds, an Agilent 1200 series equipped with a G1322A Degasser, a G1312B pump, a G1367C auto liquid sampler (ALS), a G1316B column compartment, a G1314C variable wavelength detector (VWD), and a ZORBAXSB-C18 column (50 × 3 mm, 1.8 μm) was used. The mobile phase was comprised of (A) 10% (*v*/*v*) acetic acid, and (B) 90% (*v*/*v*) acetonitrile containing 10% (*v*/*v*) acetic acid. The elution gradient was from 5% to 8% B for 5 min, from 8% to 12% B for 2 min, from 12% to 18% for 5 min, from 18% to 22% for 5 min, from 22% to 35% for 2 min, from 35% to 100% B for 2 min, 100% B for 4 min and from 100% to 5% B for 2 min with a flow rate of 1.0 mL/min. The injection volume was 2 μL and the detection wavelength was 280 nm. The column temperature was 25 °C. The MS conditions were as follows: ESI, negative ion model; nebulizer, 35 psi; dry gas flow, 10 L/min; dry gas temperature, 325 °C; scan, 100–1000 *m/z*.

Each analysis was performed in triplicate.

### 3.6. Identification and Quantification of Phenolic Compounds

The phenolic compounds in the studied red ice wines and *V. vinifera* dry red wines were mainly identified by their MS/MS information and UV absorption (anthocyanins: 525 nm; non-anthocyanin phenolics: 280 nm). For each phenolic peak, only one scan was done in every analysis. The quantification of each phenolic compounds was achieved by calculating their UV absorption peak areas in DAD (anthocyanins: 525 nm; non-anthocyanin phenolics: 280 nm). Anthocyanins were quantified by using malvidin-3-*O*-glucoside as an equivalent, and hydroxybenzoic acids, hydroxycinnamic acids, flavan-3-ols, and flavonols were quantified by using gallic acid, caffeic acid, (+)-catechin, and quercetin as equivalents, respectively.

### 3.7. Statistical Analysis

Averages and standard deviations were calculated using Microsoft Excel 2007 software. IBM SPSS Statistics 20 (IBM, New York, NY, USA) for Windows was used for statistical calculations and principal component analysis (PCA). The differences were considered to be statistically significant when *p* < 0.05. Pearson correlation analysis was performed by using MetaboAnalyst 3.0 [35].

## 4. Conclusions

In summary, Beibinghong red ice wine has unique phenolic and chromatic characteristics, especially when compared with other color-stable *V. vinifera* dry red wines. During vinification, the content of phenolic acids increased, while other flavanoids dropped. With regard to the color parameters, the changes in color intensity and red % were similar to the tendency of anthocyanins, which decreased to varying degree, while the values of hue and yellow % increased. The predominant phenolic compounds of Beibinghong red ice wine are anthocyanins, with 3,5-*O*-diglucosidic anthocyanins comprising the majority. Phenolic acids accounted for largest proportion of the non-anthocyanin compounds. In particular there were high levels of anthocyanin diglucosides, specifically malvidin-3,5-*O*-diglucoside (p16), petunidin-3,5-*O*-diglucoside (p17), delphinidin-3,5-*O*-diglucoside (p18), peonidin-3,5-*O*-diglucoside (p19), and cyanidin-3,5-*O*-diglucoside (p20), and protocatechuic acid (p24), which differentiated the Beibinghong wine from the *V*. *vinifera* wines. This suggests that the chromatic development of the Beibinghong wine is associated with the presence of these compounds. As for other phenolic compounds which are positively correlated with wine’s red %, their lower concentrations in Beibinghong icewine explains the ice wines’ weak red hue.

## Figures and Tables

**Table 1:** Sample identity and details.

Wine No.	Variety	Vintage	Wine Type	Color Stability
BH-IW13	Beibinghong	2013	Ice wine	Unstable
BH-IW14	Beibinghong	2014	Ice wine	Unstable
BH-DW14	Beibinghong	2014	Dry red wine	Unstable
ME-DW13	Merlot	2013	Dry red wine	Stable
CG-DW13	Cabernet Gernischt	2013	Dry red wine	Stable
MA-DW13	Marselan	2013	Dry red wine	Stable
CS-DW13	Cabernet Sauvignon	2013	Dry red wine	Stable

Notes: Abbreviations for Wine No.: BH-IW13: Beibinghong, icewine, 2013; BH-IW14: Beibinghong, ice wine, 2014; BH-DW14: Beibinghong, dry red wine, 2014; ME-DW13: Merlot, dry red wine, 2013; CG-DW13: Cabernet Gernischt, dry red wine, 2013; MA-DW13: Marselan, dry red wine, 2013; and CS-DW13: Cabernet Sauvignon, dry red wine, 2013.

**Table 2:** Color parameters of the seven trial wines.

Color Parameters	Wine						
	BH-IW13	BH-IW14	BH-DW14	ME-DW13	CG-DW13	MA-DW13	CS-DW13
Color Intensity (CI)	1.98 ± 0.01 ^f^	1.82 ± 0.003 ^g^	3.36 ± 0.08 ^a^	2.07 ± 0.03 ^e^	2.89 ± 0.01 d	3.3 ± 0.02 ^b^	3.19 ± 0.02 ^c^
Red %	40.88 ± 0.26 ^e^	38.73 ± 0.06 ^f^	40.78 ± 0.05 ^e^	55.81 ± 0.4 ^b^	57.13 ± 0.21 ^a^	55.04 ± 0.21 ^c^	52.19 ± 0.33 ^d^
Hue	1.07 ± 0.02 ^b^	1.12 ± 0.004 ^a^	1.03 ± 0.003 ^b^	0.61 ± 0.01 ^d^	0.56 ± 0.01 ^e^	0.59 ± 0.01 ^d,e^	0.68 ± 0.01 ^c^
Yellow %	43.63 ± 0.36 ^b^	46.41 ± 0.1 ^a^	41.85 ± 0.07 ^c^	34.03 ± 0.52 ^e^	32.24 ± 0.12 ^f^	32.42 ± 0.21 ^f^	35.31 ± 0.5 ^d^

Notes: Each analysis was performed in triplicate. Different letters in each row indicate the significant differences at *p* < 0.05. All data were expressed as the mean value ± the standard deviation. Abbreviations for Wine No.: BH-IW13: Beibinghong, icewine, 2013; BH-IW14: Beibinghong, icewine, 2014; BH-DW14: Beibinghong, dry red wine, 2014; ME-DW13: Merlot, dry red wine, 2013; CG-DW13: Cabernet Gernischt, dry red wine, 2013; MA-DW13: Marselan, dry red wine, 2013; and CS-DW13: Cabernet Sauvignon, dry red wine, 2013.

**Table 3:** The phenolic compound content (mg/L) of the seven trial wines.

Phenolic Compounds	No.	MS/MS Information	Wine						
			BH-IW13	BH-IW14	BH-DW14	ME-DW13	CG-DW13	MA-DW13	CS-DW13
Malvidin-3-*O*-glucoside	p1	493 (331)	37.71 ± 1.18 ^d^	10.53 ± 0.1 ^f^	11.16 ± 0.08 ^e^	63.78 ± 1.47 ^c^	105.98 ± 0.91 ^a^	80.77 ± 0.16 ^b^	61.77 ± 0.08 ^c^
Petunidin-3-*O*-glucoside	p2	479 (317)	14.01 ± 0.63 ^d^	3.01 ± 0.01 ^g^	6.11 ± 0.01 ^f^	26.26 ± 0.13 ^b^	38.1 ± 0.04 ^a^	10.6 ± 0.16 ^e^	20.05 ± 0.14 ^c^
Delphinidin-3-*O*-glucoside	p3	465 (303)	5.35 ± 0.23 ^d^	0.92 ± 0.04 ^g^	1.57 ± 0.02 ^f^	16.46 ± 0.19 ^b^	23.71 ± 0.76 ^a^	4 ± 0.1 ^e^	13.61 ± 0.5 ^c^
Peonidin-3-*O*-glucoside	p4	463 (301)	1.76 ± 0.11 ^e^	Tr	1.34 ± 0.01 ^f^	14.84 ± 0.03 ^b^	15.7 ± 0.27 ^a^	3.42 ± 0.02 ^d^	6.32 ± 0.03 ^c^
Cyanidin-3-*O*-glucoside	p5	449 (287)	0.6 ± 0.02 ^e^	Tr	1.26 ± 0.01 ^d^	4.69 ± 0.16 ^a^	4.3 ± 0.03 ^b^	Tr	1.7 ± 0.01 ^c^
Malvidin-3-*O*-acetylglucoside	p6	535 (331)	5.31 ± 0.2 ^e^	1.1 ± 0.04 ^g^	2.79 ± 0.02 ^f^	28.05 ± 0.16 ^d^	46.4 ± 0.2 ^b^	48.54 ± 0.38 ^a^	30.55 ± 0.24 ^c^
Petunidin-3-*O*-acetylglucoside	p7	521 (317)	2.89 ± 0.19 ^e^	Tr	1.21 ± 0.04 ^f^	15.45 ± 0.25 ^b^	22 ± 0.12 ^a^	7.08 ± 0.39 ^d^	10.98 ± 0.05 ^c^
Delphinidin-3-*O*-acetylglucoside	p8	507 (303)	0.89 ± 0.03 ^e^	Tr	Tr	7.23 ± 0.25 ^b^	10.02 ± 0.24 ^a^	2 ± 0.02 ^d^	5.36 ± 0.11 ^c^
Peonidin-3-*O*-acetylglucoside	p9	505 (301)	Tr *	Tr	Tr	6.38 ± 0.13 ^b^	7.32 ± 0.16 ^a^	2.45 ± 0.1 ^d^	3.28 ± 0.03 ^c^
Cyanidin-3-*O*-acetylglucoside	p10	491 (287)	0.54 ± 0.01 ^c^	Tr	Tr	2.85 ± 0.04 ^a^	2.89 ± 0.02 ^a^	Tr	1.11 ± 0.01 ^b^
Malvidin-3-*O*-coumaroylglucoside	p11	639 (331)	1.93 ± 0.02 ^e^	Tr	0.61 ± 0.01 ^f^	11.05 ± 0.05 ^d^	25.64 ± 0.03 ^a^	18.99 ± 0.31 ^b^	13.63 ± 0.09 ^c^
Petunidin-3-*O*-coumaroylglucoside	p12	625 (317)	0.44 ± 0.02 ^e^	Tr	Tr	4.16 ± 0.24 ^b^	8.12 ± 0.16 ^a^	2.05 ± 0.08 ^d^	2.78 ± 0.02 ^c^
Delphinidin-3-*O*-coumaroylglucoside	p13	611 (303)	Tr	Tr	Tr	2.07 ± 0.01 ^b^	3.54 ± 0.02 ^a^	0.77 ± 0.01 ^d^	1.44 ± 0.04 ^c^
Peonidin-3-*O*-coumaroylglucoside	p14	609 (301)	Tr	Tr	Tr	3.7 ± 0.04 ^b^	5.56 ± 0.01 ^a^	1.55 ± 0.05 ^d^	2.02 ± 0.01 ^c^
Cyanidin-3-*O*-coumaroylglucoside	p15	595 (287)	Tr	Tr	Tr	1.46 ± 0.01 ^b^	1.65 ± 0.02 ^a^	Tr	Tr
Malvidin-3,5-*O*-diglucoside	p16	655 (331)	74.21 ± 1.03 ^c^	83.86 ± 0.4 ^b^	94.98 ± 1.67 ^a^	Nd	Nd	Nd	Nd
Petunidin-3,5-*O*-diglucoside	p17	641 (317)	5.47 ± 0.39 ^b^	3.88 ± 0.08 ^c^	12.63 ± 0.35 ^a^	Nd	Nd	Nd	Nd
Delphinidin-3,5-*O*-diglucoside	p18	627 (303)	2.33 ± 0.22 ^b^	1.31 ± 0.02 ^c^	2.43 ± 0.08 ^a^	Nd	Nd	Nd	Nd
Peonidin-3,5-*O*-diglucoside	p19	625 (301)	12.25 ± 0.33 ^b^	4.48 ± 0.1 ^c^	18.17 ± 0.22 ^a^	Nd	Nd	Nd	Nd
Cyanidin-3,5-*O*-diglucoside	p20	611 (287)	2.63 ± 0.14 ^b^	0.96 ± 0.02 ^c^	6.14 ± 0.18 ^a^	Nd	Nd	Nd	Nd
Pelargonidin-3,5-*O*-diglucoside	p21	595 (271)	Nd **	Nd	Tr	Nd	Nd	Nd	Nd
Anthocyanin content			168.32	110.05	160	208.43	320.93	182.22	174.6
Anthocyanin proportion (%)			93.03	92.06	59.29	29.81	37.67	28.77	23.76
2-hydroxybenzoic acid	p22	137 (93)	Tr	Tr	0.84 ± 0.01 ^a^	0.28 ± 0.02 ^c^	0.48 ± 0.06 ^b^	Tr	Tr
Gallic acid	p23	169 (125)	0.84 ± 0.09 ^f^	Tr	40.09 ± 0.18 ^e^	57.66 ± 0.47 ^c^	75.56 ± 0.59 ^a^	46.02 ± 0.15 ^d^	62.81 ± 0.04 ^b^
Protocatechuic acid	p24	153 (109)	5.24 ± 0.25 ^c^	8 ± 0.63 ^b^	12.16 ± 0.01 ^a^	1.67 ± 0.03 ^e^	2.18 ± 0.03 ^d^	0.7 ± 0.02 ^f^	0.99 ± 0.04 ^f^
Vanillic acid	p25	167 (152)	2.37 ± 0.18 ^e^	Nd	2.47 ± 0.05 ^e^	3.71 ± 0.12 ^c^	4.55 ± 0.04 ^b^	3.1 ± 0.04 ^d^	5.17 ± 0.06 ^a^
Syringic acid	p26	301 (229)	Nd	0.97 ± 0.01 ^e^	Nd	2.7 ± 0.01 ^d^	3.42 ± 0.03 ^c^	6.41 ± 0.03 ^b^	8.44 ± 0.01 ^a^
4-hydroxycinnamic acid	p27	163 (119)	0.26 ± 0.02 ^e^	Nd	4.35 ± 0.02 ^b^	1.48 ± 0.02 ^d^	5.45 ± 0.01 ^a^	3.19 ± 0.06 ^c^	1.41 ± 0.06 ^d^
Caffeic acid	p28	179 (135)	3.3 ± 0.19 ^d^	Tr	6.97 ± 0.03 ^a^	Tr	2.88 ± 0.02 ^e^	11.2 ± 0.1 ^b^	4.59 ± 0.58 ^c^
Gentisic acid	p29	153 (108)	Nd	Nd	Tr	Nd	Tr	Nd	Nd
Ferulic acid	p30	193 (134)	Tr	Nd	0.24 ± 0.03 ^d^	0.27 ± 0.03 ^c^	0.33 ± 0.01 ^bc^	0.45 ± 0.01 ^a^	0.33 ± 0.01 ^bc^
Phenolic acids content			12.01	8.97	67.12	67.77	94.85	71.07	83.74
Phenolic acids proportion (%)			6.64	7.5	24.87	9.69	11.13	11.22	11.4
(+)-Catechin	p31	289 (123)	Nd	Nd	6.5 ± 0.1 ^e^	81.83 ± 0.84 ^c^	75.7 ± 0.19 ^d^	90.02 ± 0.22 ^b^	114.26 ± 0.1 ^a^
(−)-Epicatechin	p32	289 (123)	Nd	Nd	7.82 ± 0.19 ^e^	116.39 ± 0.9 ^b^	95.44 ± 0.04 ^d^	107.98 ± 0.2 ^c^	137.77 ± 0.2 ^a^
Gallocatechin	p33	305 (125)	Nd	Nd	0.26 ± 0.01 ^e^	24.8 ± 0.44 ^b^	30.01 ± 0.09 ^a^	9.89 ± 0.02 ^d^	13.3 ± 0.03 ^c^
Epigallocatechin	p34	305 (125)	Nd	Nd	Tr	Nd	Nd	0.45 ± 0.01 ^a^	Nd
Procyanin B1	p35	577 (407)	Tr	Nd	2.13 ± 0.04 ^e^	46.13 ± 0.33 ^b^	47.18 ± 0.02 ^a^	42.14 ± 0.31 ^d^	44.63 ± 0.14 ^c^
Procyanin B2	p36	577 (407)	Tr	Nd	0.91 ± 0.11 ^e^	22.7 ± 0.06 ^d^	24.46 ± 0.01 ^c^	26.33 ± 0.35 ^b^	32.18 ± 0.02 ^a^
Procyanin C1	p37	865 (407)	Nd	Nd	Nd	13.98 ± 0.34 ^c^	13 ± 0.03 ^d^	15.9 ± 0.2 ^b^	19.48 ± 0.04 ^a^
Flavan-3-ols content			Nd	Nd	17.63	305.83	285.79	292.71	361.62
Flavan-3-ols proportion (%)			Nd	Nd	6.53	43.75	33.55	46.21	49.21
Kaempferol	p38	285 (117)	Nd	Nd	Nd	5.56 ± 0.29 ^b^	8.86 ± 0.14 ^a^	0.69 ± 0.03 ^d^	1.18 ± 0.03 ^c^
Quercetin	p39	301 (151)	Nd	Nd	3.66 ± 0.06 ^e^	45.77 ± 0.23 ^b^	73.75 ± 0.34 ^a^	11.08 ± 0.04 ^d^	15.45 ± 0.15 ^c^
Dihydrokaempferol	p40	287 (259)	Nd	Tr	0.97 ± 0.02 ^c^	1.37 ± 0.03 ^a^	1.23 ± 0.04 ^b^	0.69 ± 0.02 ^e^	0.83 ± 0.02 ^d^
Dihydroquercetin	p41	303 (125)	Nd	Tr	3.41 ± 0.03 ^a^	1.94 ± 0.15 ^c^	2.28 ± 0.02 ^b^	1.01 ± 0.01 ^d^	1.17 ± 0.03 ^d^
Isorhamnetin	p42	315 (300)	Nd	Nd	Tr	5.47 ± 0.16 ^b^	10.16 ± 0.16 ^a^	2.19 ± 0.04 ^d^	3.15 ± 0.01 ^c^
Myricetin	p43	317 (151)	Nd	Nd	0.94 ± 0.01 ^e^	3.52 ± 0.2 ^b^	8.66 ± 0.05 ^a^	2.11 ± 0.02 ^d^	2.47 ± 0.02 ^c^
Kaempferol-3-*O*-glucoside	p44	447 (255)	Nd	Nd	Tr	Tr	Tr	0.62 ± 0.02 ^b^	1.06 ± 0.04 ^a^
Kaempferol-3-*O*-galactoside	p45	447 (255)	Nd	Nd	Tr	0.65 ± 0.05 ^a^	0.37 ± 0.02 ^b^	Tr	Tr
Quercetin-3-*O*-glucoside	p46	463 (300)	Tr	Nd	0.19 ± 0.02 ^d^	10.31 ± 0.38 ^b^	Tr	8.81 ± 0.03 ^c^	12.57 ± 0.02 ^a^
Quercetin-3-*O*-galactoside	p47	463 (300)	0.36 ± 0.02 ^f^	Nd	0.9 ± 0.02 ^e^	4.03 ± 0.06 ^a^	3.75 ± 0.02 ^b^	2.1 ± 0.03 ^d^	2.8 ± 0.04 ^c^
Quercetin-3-*O*-glucuronide	p48	477 (301)	0.24 ± 0.02 ^f^	Nd	8.74 ± 0.01 ^e^	15.29 ± 0.3 ^c^	17.28 ± 0.12 ^a^	14.01 ± 0.05 ^d^	15.98 ± 0.03 ^b^
Isorhamnetin-3-*O*-glucoside	p49	477 (314)	Nd	Nd	Tr	4.6 ± 0.33 ^b^	1.79 ± 0.01 ^d^	3.16 ± 0.53 ^c^	6.19 ± 0.02 ^a^
Myricetin-3-*O*-glucoside	p50	479 (316)	Tr	Nd	5.35 ± 0.04 ^d^	14.55 ± 0.05 ^c^	14.46 ± 0.03 ^c^	26.6 ± 0.34 ^b^	33.07 ± 0.06 ^a^
Myricetin-3-*O*-galactoside	p51	479 (316)	Nd	Nd	Tr	1.75 ± 0.07 ^d^	3.25 ± 0.04 ^b^	2.18 ± 0.01 ^c^	3.75 ±0.08 ^a^
Syringetin	p52	345 (315)	Nd	Nd	Tr	Tr	Tr	0.38 ± 0.01 ^b^	0.53 ± 0.02 ^a^
Syringetin-3-*O*-glucoside	p53	507 (344)	Nd	0.52 ± 0.04 ^e^	0.96 ± 0.01 ^f^	3.17 ± 0.05 ^d^	4.26 ± 0.02 ^c^	11.32 ± 0.05 ^b^	14.13 ± 0.04 ^a^
Syringetin-3-*O*-galactoside	p54	507 (344)	Nd	Nd	Tr	Tr	0.23 ± 0.01 ^c^	0.44 ± 0.01 ^b^	0.55 ± 0.04 ^a^
Flavonols content			0.6	0.52	25.12	117.08	150.33	87.39	114.88
Flavonols proportion (%)			0.33	0.44	9.3	16.75	17.65	13.8	15.63
Total polyphenol			180.93	119.54	269.86	699.11	851.9	633.39	734.84

Note: Each analysis was performed in triplicate. MS/MS information included the precursor ion (in front of the bracket) and the major fragment ion (in the bracket) of each phenolic compound. Different letters in each row indicate the significant differences at *p* < 0.05. All data were expressed as the mean value ± the standard deviation. Abbreviations for Wine No.: BH-IW13: Beibinghong, icewine, 2013; BH-IW14: Beibinghong, icewine, 2014; BH-DW14: Beibinghong, dry red wine, 2014; ME-DW13: Merlot, dry red wine, 2013; CG-DW13: Cabernet Gernischt, dry red wine, 2013; MA-DW13: Marselan, dry red wine, 2013; CS-DW13: Cabernet Sauvignon, dry red wine, 2013. The content of each anthocyanin, hydroxybenzoic acid, hydroxycinnamic acid, flavan-3-ol and flavonol in this table is equivalent to that of malvidin-3-*O*-glucoside, gallic acid, caffeic acid, (+)-catechin and quercetin, respectively. The detection limit (LOD) of malvidin-3-*O*-glucoside, gallic acid, caffeic acid, (+)-catechin and quercetin in methanol is 0.13 mg/L, 0.065 mg/L, 0.05 mg/L, 0.075 mg/L and 0.05 mg/L, respectively. The quantification limit (LOQ) of malvidin-3-*O*-glucoside gallic acid, caffeic acid, (+)-catechin and quercetin in methanol is 0.4 mg/L, 0.16 mg/L, 0.2 mg/L, 0.24 mg/L and 0.17 mg/L, respectively. The calculations for LOD and LOQ were based on the standard deviation of the response (R) and the slope (S) of the calibration curve at the levels approaching the limits according to equation R/S = 3 (LOD) and R/S = 10 (LOQ). The determination of LOD and LOQ were performed in triplicate. * Tr indicates trace (phenolic concentration which was below LOQ but above LOD). ** Nd indicates not detected (phenolic concentration which was below LOD).
